# Aortic pressure-velocity loop: a potential tool for assessing risk associated with midsystolic forward compression waves in patients with chronic atherosclerotic coronary artery disease

**DOI:** 10.1016/j.ahjo.2026.100787

**Published:** 2026-04-17

**Authors:** Shizuo Hanya, Hajime Yamakage, Yukiyoshi Kondoh, Motoaki Sugawara

**Affiliations:** a Department of Cardiovascular Science, The Heart Institute of Japan, Tokyo Women's Medical University, 8-1 Kawada-cho, Shinjuku-ku, Tokyo, 162-8666, Japan; b Department of Endocrinology, Metabolism, and Hypertension Research, Clinical Research Institute, NHO Kyoto Medical Center, 1-1 Mukaihata-cho, Fukakusa, Fushimi-ku, Kyoto, Japan

**Keywords:** Blood inertia, Local aortic pulse wave speed, Wave reflection, Ventriculo-arterial interaction, Multisensor catheter, Wave intensity analysis

## Abstract

**Study objective:**

To evaluate whether impaired ventriculo-aretrial interaction characterizes chronic ischemic heart disease (CIHD) caused by stable chronic obstructive coronary artery disease (CAD), using wave intensity and aortic pressure–velocity (PU) loop analyses.

**Design:**

Retrospective observational study.

**Setting:**

Single-center invasive hemodynamic assessment using high-fidelity multisensor catheters.

**Participants:**

Thirty-one patients with CIHD, including 21 with chronic atherosclerotic CAD and 10 with non-CAD CIHD.

**Interventions:**

None.

**Main outcome measure(s):**

Wave intensity–derived indices of ventriculo-arterial interaction, including the initial PU-loop slope (S1), second ascending slope (S2), backward compression wave (BCW), midsystolic forward compression wave (m-FCW), and their relative magnitudes. Discriminatory ability for CAD was assessed using receiver operating characteristic (ROC) curve analysis.

**Results:**

In CAD patients, midsystolic flow was supported by a previously unreported m-FCW that emerged in response to an early-onset BCW during ventricular acceleration, reflected by higher S2 values. In contrast, midsystolic flow in non-CAD CIHD patients was primarily maintained by blood inertia and lower S1 values. S2 strongly correlated with the relative magnitude of m-FCW (*r* = −0.936, *P* < 0.001). The relative magnitude of m-FCW showed greater discriminatory ability for CAD than BCW (AUC 0.976 vs. 0.771). The S2:S1 ratio correlated closely with the relative magnitude of m-FCW and demonstrated the highest AUC (0.986).

**Conclusions:**

Chronic atherosclerotic CAD is characterized by impaired ventriculo-aortic interaction, with midsystolic flow dependent on compensatory m-FCW rather than blood inertia. Aortic PU-loop parameters, particularly the S2:S1 ratio, may provide a potential diagnostic marker for distinguishing chronic atherosclerotic CAD from other forms of CIHD.

## Highlights

• Early-onset wave reflection opposing blood inertia increases cardiovascular risk.
• The aortic pressure-velocity loop's initial linear slope (S1) reflects blood inertia.
• Reduced inertia is indicated by a midsystolic forward compression wave (m-FCW).
• The magnitude of the m-FCW is reflected in the loop's second ascending slope (S2).
• The S2:S1 ratio reflects cardiovascular risk as a marker of the blood inertia effect.

## Graphical abstract

**Figure:**
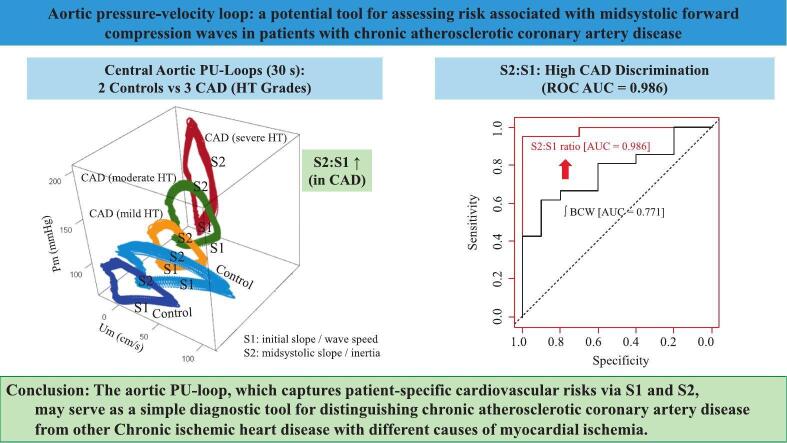

## 1 Introduction

As well expressed by the axiom that “man is as old as his arteries”, healthy longevity is closely linked to vascular health. Even in the 21st century, atherosclerotic cardiovascular diseases still remain the leading cause of death globally. In patients with chronic atherosclerotic coronary artery disease (CAD), atherosclerosis-induced stiffening of the coronary and systemic arteries can alter cardiac performance and wave reflections, altering ventriculo-arterial interaction [1]. Altered ventriculo-arterial interaction may, in turn, further impair coronary circulation and cardiac performance, creating a vicious cycle. Thus, early detection of altered ventriculo-arterial interaction in the central aorta may help distinguish high-risk chronic atherosclerotic CAD from other nonatherosclerotic chronic ischemic heart disease (CIHD). Clinical assessments of these properties should be conducted in the central aorta because it is the vessel most affected by stiffening and provides direct insight into impaired left ventricular output [2], [3], [4].

Simultaneous, high-fidelity measurement of pressure (P) and flow velocity (U) at the same site in the central aorta is essential for detailed analysis of ventriculo-arterial interaction. Multisensor catheters allow for such measurement and have enabled several key studies of ventriculo-arterial interaction [5], [6]. Using a multisensor catheter, Parker et al. introduced wave intensity analysis (WIA), which allows forward- and backward-traveling waves to be related to specific time points for assessing ventriculo-arterial interaction in the central aorta [7]. Separation of P, U, and wave intensity into forward and backward components in WIA requires estimation of pulse wave speed (PWS). The aortic pressure-velocity loop (PU-loop) was introduced as a tool to determine local aortic PWS [8].

As the clinical application of WIA largely coincided with a shift from invasive to noninvasive techniques, limited WIA findings by multisensor catheter measurements in the human central aorta are available [9]. The aortic PU-loop has previously been used for intraoperative monitoring of left ventricular afterload, based on the angle formed between the baseline and the line connecting the origin of the loop to the point of maximum P [10]. The PU-loop in the main pulmonary artery has been also reported to have potential for assessing the severity of pulmonary hypertension [11].

Recently, noninvasive measurement of aortic diameter (D) has been proposed as a surrogate of P, and local aortic PWS has been calculated using the aortic DU-loop derived from phase contrast magnetic resonance imaging (MRI) [12]. In this context, we propose the aortic PU-loop, obtained by simply plotting P against U, as an intuitive tool for identifying chronic atherosclerotic CAD patients at high cardiovascular risk among those with non-atherosclerotic CIHD [13], [14]. This method may enable reliable, noninvasive risk stratification in patients with stable atherosclerotic CAD.

## 2 Materials and methods

### 2.1 Study population

We retrospectively reviewed records of 31 consecutive patients with chest pain and a left ventricular ejection fraction greater than 55% who underwent diagnostic coronary angiography under the diagnosis of chronic coronary syndromes at Tokyo Women's Medical University and its affiliated hospital. Simultaneous, high-fidelity multisensor catheter measurements of P and U in the central aorta were obtained. Exclusion criteria included heart failure, regional wall motion abnormalities, and prominent valvular diseases.

All procedures were performed in accordance with the principles of the Declaration of Helsinki, and all patients gave written informed consent. The study was approved by the Ethics Committee of Tokyo Women's Medical University and was performed in accordance with institutional guidelines in effect at the time.

### 2.2 Cardiac catheterization

Patients continued all medications prescribed by their treating physicians and were studied in a basal state. Custom-designed left-heart VPC-684 multisensor catheters (Millar Inc., Houston, TX, USA) were used. These catheters are equipped with a solid-state pressure sensor and an electromagnetic flow velocity sensor positioned laterally at the same site, 5 cm proximal to the catheter tip [15]. After diagnostic coronary angiography, the catheter was inserted via the right or left femoral artery and advanced retrogradely across the aortic valve, with the tip positioned in the left ventricular cavity to maximize stability. The positions of the P and U sensors were carefully adjusted to obtain high-fidelity recordings. Because catheter position critically affects measurement accuracy, particularly for U, special care was taken to align the U sensor parallel to blood flow within the aorta. Catheter movement was minimized by careful adjustment to ensure an accurate peak U signal on the polygraph.

### 2.3 Data recording, signal processing, and data analysis

All analog outputs from the VPC-684 catheter were recorded at a sampling rate of 500 Hz, stored digitally, and fed into an MP100WS (BIOPAC Systems, Inc., Santa Barbara, CA, USA) to graph the wave intensity (WI) waveform and aortic PU-loop. A 62.5-Hz low-pass filter was applied during analysis to remove high-frequency electrical interference that could interfere with differentiation of P and U. The velocimetric hardware-related time delay was calibrated in advance using a specially designed apparatus incorporating a displacement meter and was corrected before data analysis [15]. The time derivatives of P and U were obtained every 2 milliseconds. Because continuous ensemble averaging alone made it difficult to analyze the relationships between the waveforms and their PU-loops in detail, P and U signals were ensemble-averaged over five beats.

### 2.4 Calculation of WI in the central aorta

#### 2.4.1 Overview of WIA

Wave intensity (net-WI) is defined as dP • dU [7]. A positive value indicates that waves traveling toward the periphery (forward waves; WI_f_) are predominant; this occurs when P and U increase or decrease simultaneously. A negative value indicates that waves reflected from the periphery (backward waves; WI_b_) are predominant; this occurs when changes in P and U are reciprocal.

Waves are further classified based on their effect on pressure: waves that increase P are defined as compression waves, whereas those that decrease P are defined as decompression waves. A compression wave traveling from the left ventricle in early ejection contributes to the acceleration of blood in the central aorta and corresponds to the first positive predominant WI_f_. This wave may be regarded as the “initial ventricular impulse” described by Rushmer [16] and may be useful for assessing left ventricular performance as an impulse generator. Thus, WI is a hemodynamic variable that may provide information about ventriculo-arterial interaction and the fluid dynamic consequences for cardiac output during myocardial contraction and relaxation.

To analyze PU-loop characteristics, we performed WIA as described elsewhere [17] and as shown in Eq. (1):

(1) $$\mathrm{net}-\mathrm{WI}=\left(\mathrm{dP}/\mathrm{dt}\right)•\left(\mathrm{dU}/\mathrm{dt}\right)$$

where dP and dU are the measured changes in P and U, respectively, at 2-millisecond intervals.

The forward component (WI_f_) and backward component (WI_b_) of net-WI were calculated using Eqs. (2), (3) [17]:

$$\mathrm{net}-\mathrm{WI}={\mathrm{WI}}_{f}+{\mathrm{WI}}_{b}$$

(2) $${\mathrm{WI}}_{f}=\left({\mathrm{dP}}_{f}/\mathrm{dt}\right)•\left({\mathrm{dU}}_{f}/\mathrm{dt}\right)={\left({\mathrm{dP}}_{f}/\mathrm{dt}\right)}^{2}/\rho •\mathrm{PWS}$$

(3) $${\mathrm{WI}}_{b}=\left({\mathrm{dP}}_{b}/\mathrm{dt}\right)•\left({\mathrm{dU}}_{b/}\mathrm{dt}\right)=-{\left({\mathrm{dP}}_{b}/\mathrm{dt}\right)}^{2}/\rho •\mathrm{PWS}$$

where P_f_ and U_f_ are the forward-traveling components of P and U; P_b_ and U_b_ are the backward-traveling components of P and U; ρ is the blood density; and PWS is the local aortic pulse wave speed, calculated using the water hammer equation [18].

P_f_ and P_b_ were calculated using Eqs. (4), (5):

(4) $$P_{f}=½\;\left(P+\rho •\mathrm{PWS}•U\right)$$

(5) $$P_{b}=½\;\left(P-\rho •\mathrm{PWS}•U\right)$$

#### 2.4.2 PU-loop characteristics based on WIA

**Fig. 1:**
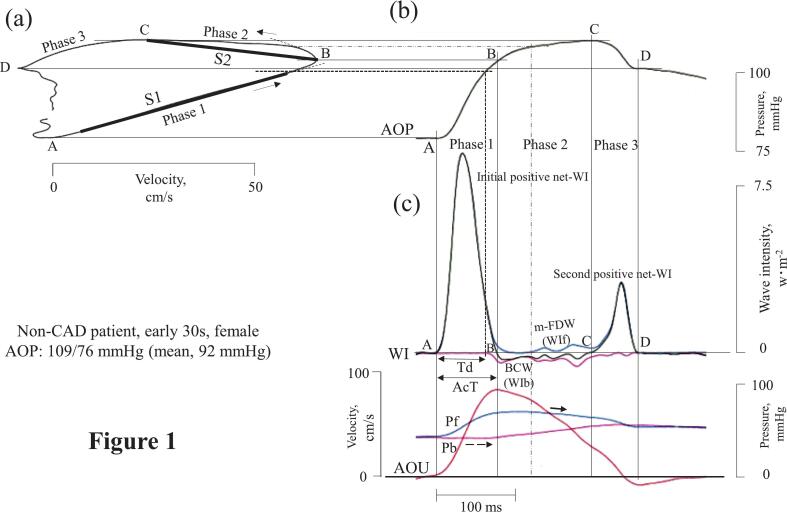
Relationship between aortic pressure-velocity loop and wave intensity analysis in a representative female control patient without hypertension The aortic pressure-velocity loop is shown in (a); the arrows indicate the direction of the loop. In (b), the pressure is plotted against time to translate each point of the loop to the time. Part (c) shows the corresponding wave intensity analysis, aortic flow velocity, and backward- and forward-traveling aortic pressure components. AcT, ventricular acceleration time; AOP, aortic pressure; AOU, aortic flow velocity; BCW, backward compression wave; m-FDW, midsystolic forward decompression wave; Pb, backward-traveling component of AOP; Pf, forward-traveling component of AOP; S1, initial linear A-B ascending slope of the aortic pressure-velocity loop until the onset of backward wave; S2, slope of the pressure-velocity loop's second B-C ascending limb from the peak velocity point (B) to the peak aortic pressure point (C); Td, time between the beginning of the initial positive net wave intensity and the beginning of BCW; WI, wave intensity; WIb, backward-traveling component of WI; WIf, forward-traveling component of WI.

The aortic PU-loop is characterized by four points, three phases, and two markers (Fig. 1a).

-Points-

Point A: Onset of the initial positive net-WI.

Point B: End point of the initial positive net-WI, which corresponds to the point of peak aortic flow velocity.

Point C: Onset of the second positive net-WI, which corresponds to the point of peak aortic pressure.

Point D: End point of the second positive net-WI.

-Phases and markers-

*Phase 1*: The A-B line of the loop corresponds to the initial positive net-WI, which represents the initial left ventricular acceleration and acceleration time (AcT).

Marker 1: S1 is the initial linear slope of the A-B line, up to the onset of the first backward wave (bold dashed line), and is used to calculate local aortic PWS.

*Phase 2*: The B-C ascending limb corresponds to midsystole, between the end of the first and onset of the second positive net-WI waves.

Marker 2: S2 is the slope of the B-C ascending limb from the point of peak aortic flow velocity (B) to the point of peak aortic pressure (C).

*Phase 3*: The C-D descending limb corresponds to the second positive net-WI wave.

Because the measurement of interest was the increase in P, we used the absolute values of S1 and S2 to calculate the following parameters:

S1 + S2: sum of the absolute values of S1 and S2

S2:S1 ratio: absolute value of S2 divided by absolute value of S1

#### 2.4.3 Quantification of waves

The relative magnitude of each pulse wave was quantified by dividing its cumulative WI by that of the initial positive net-WI (∫ initial net-WI).

#### 2.4.4 Measurement of BCW onset time

The relative onset time of the backward compression wave (BCW) was expressed as %Td and calculated using Eq. (6):

(6) %Td=Td/AcT

where Td is the time between the onset of the initial positive net-WI and the onset of the BCW (Fig. 1a).

### 2.5 Statistical analysis

This study was exploratory in nature, and the relatively small sample size (*n* = 31) was determined by the number of consecutive patients who met the eligibility criteria during the study period. Therefore, no formal sample size calculation was performed. The statistical analyses were intended to generate hypotheses and provide preliminary insights rather than to yield definitive conclusions. Accordingly, no correction for multiple testing was applied.

Continuous data are expressed as medians and interquartile ranges (25th percentile, 75th percentile). For group comparisons, the Mann-Whitney *U* test was used for continuous variables, and Fisher's exact test was used for categorical variables. For continuous wave parameters compared between groups (CAD vs. non-CAD), effect sizes were additionally calculated as *r* for the Mann–Whitney U test. The ability of the variables to discriminate patients with CAD was assessed by receiver operating characteristic (ROC) curve analysis. The area under the curve (AUC) was calculated as the discriminatory ability index, and the optimal cutoff was defined as the point on the ROC curve that maximized the Youden index (sensitivity + specificity −1).

Spearman's correlation coefficients were calculated between variables obtained from WIA and the PU-loop. All statistical analyses were performed using R software (version 4.2.2; R Foundation for Statistical Computing, Vienna, Austria). A *P* value <0.05 was considered statistically significant.

## 3 Results

### 3.1 Patient characteristics

Apparent atherosclerotic chronic obstructive CAD was defined as >50% stenosis in one or more epicardial coronary arteries, as confirmed on coronary angiography by three radiologists. We divided the 31 CIHD patients with at least one year of chest pain controlled by medications and suspected myocardial ischemia on an electrocardiogram into two groups: CAD (*n* = 21) and non-CAD (*n* = 10) (Table 1). In the 21 patients with CAD, 15 had one-vessel disease (71.4%) and 6 had two-vessel disease (28.6%). Patients with three-vessel disease were excluded from the study to reduce the invasive burden. Arteriosclerosis obliterans (ASO, *n* = 5) was observed only in the CAD group. Minimum and mean aortic pressure (AOP) did not significantly differ between the two groups. Age and peak AOP were significantly higher (*P* = 0.002 and <0.001, respectively) and hypertension (HT, *n* = 19) was significantly more common in the CAD group (*P* = 0.002). No significant intergroup differences were observed in sex distribution or incidence of atrial fibrillation.

**Table 1:** Patient characteristics and comorbidities.

Characteristic	Overall (*n* = 31)	Non-CAD group (*n* = 10)	CAD group (*n* = 21)	*P* value
Sex				0.121
Male	17 (54.8%)	3 (30.0%)	14 (66.7%)	
Female	14 (45.2%)	7 (70.0%)	7 (33.3%)	
Age, y	61.0 [53.0, 71.0]	48.0 [36.3, 65.8]	65.0 [59.0, 74.0]	**0.002**^a^
Peak AOP, mmHg	128 [117, 141]	117 [115, 122]	135 [128, 151]	**<0.001**^a^
Minimum AOP, mmHg	67 [62, 73]	69 [65, 73]	65 [58, 74]	0.370
Mean AOP, mmHg	91 [87, 97]	91 [88, 94]	94 [85, 103]	0.512
Pulse pressure, mmHg	60 [50, 77]	50 [47, 55]	72 [59, 81]	**<0.001**^a^
Hypertension	19 (61.3%)	2 (20.0%)	17 (81.0%)	**0.002**^a^
Atrial fibrillation	6 (19.4%)	2 (20.0%)	4 (19.0%)	0.999
CAD	21 (67.7%)	0 (0.0%)	21 (100.0%)	–
ASO	5 (16.1%)	0 (0.0%)	5 (23.8%)	–
CAD & ASO	3 (9.7%)	0 (0.0%)	3 (14.3%)	–

Data are shown as median [interquartile range: 25th percentile, 75th percentile] or n (%). *P* value: Mann-Whitney U test or Fisher's exact test.

AOP, aortic pressure; ASO, arteriosclerosis obliterans; CAD, coronary artery disease.

a Significant *P* values (*P* < 0.05) are written in bold.

### 3.2 Representative aortic PU-loops and WIA

**Fig. 2:**
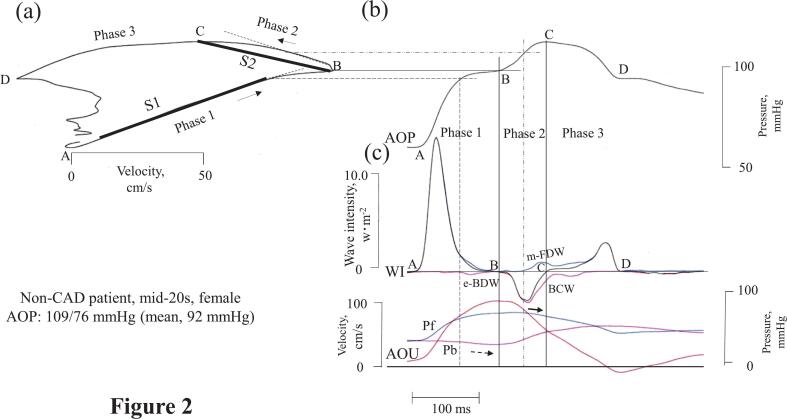
Relationship between aortic pressure-velocity loop and wave intensity analysis in the youngest control patient The aortic pressure-velocity loop is shown in (a); the arrows indicate the direction of the loop. In (b), pressure is plotted against time to translate each point of the loop to the time. In (c), wave intensity, flow velocity, and the backward- and forward-traveling aortic pressure components, which are matched to the pressure times in (b), are shown. e-BDW, early backward decompression wave. Other abbreviations are the same as in Fig. 1.

Fig. 1, Fig. 2 show the relationship between WIA and aortic PU-loop in two non-CAD patients. In both cases, a small late midsystolic forward decompression wave (m-FDW) and a decrease in P_f_ in phase 2 (bold solid arrow) were observed. At this point, the m-FDW flattened the ascending B-C limb (irregular dashed line). In the youngest non-CAD patient (Fig. 2), a small early-onset backward decompression wave (e-BDW) in phase 1 caused the loop's A-B line to bend downwards, along with a decrease in P_b_ (dashed arrow). Although the cumulative WI of the e-BDW was too small to quantify, the downward bending of the A-B slope was evident.

**Fig. 3:**
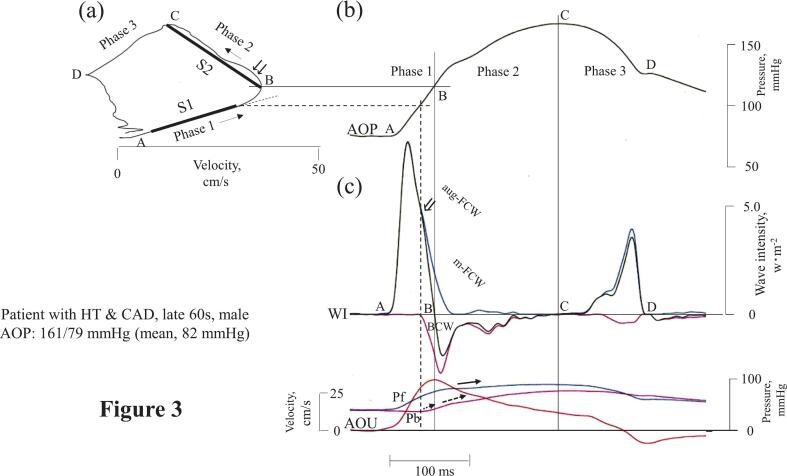
Aortic pressure-velocity loop and wave intensity relationship in a typical CAD patient with HT The aortic PU-loop is shown in (a); the arrows indicate the direction of the loop. In (b), pressure is plotted against time to translate each point of the loop to the time. Wave intensity, flow velocity, and the backward- and forward-traveling aortic pressure components, which are matched to the respective time of P in (b), are shown in (c). CAD, coronary artery disease; HT, hypertension; m-FCW, midsystolic forward compression wave. Other abbreviations are the same as in Fig. 1.

In a hypertensive patient with CAD (Fig. 3), the early onset of the BCW in phase 1 coincided with an augmented forward compression wave (aug-FCW) that originated from the first positive net-WI (⇓). This wave bent the loop's A-B slope upward toward the peak velocity point B and caused an increase in P_b_ (fine dashed arrow). In this patient, S1 was much shorter than in the aforementioned non-CAD patients because of the earlier onset of the BCW. The aug-FCW continued into phase 2, where we termed it the midsystolic forward compression wave (m-FCW). This wave made the loop's B-C ascending limb steeper and convex toward the U axis (⇊) and was accompanied by increases in both P_f_ (bold arrow) and P_b_ (bold dashed arrow) associated with the BCW.

### 3.3 Comparison of aortic PU-loops and WIA findings in one non-CAD patient and three CAD patients with varying HT severity

**Fig. 4:**
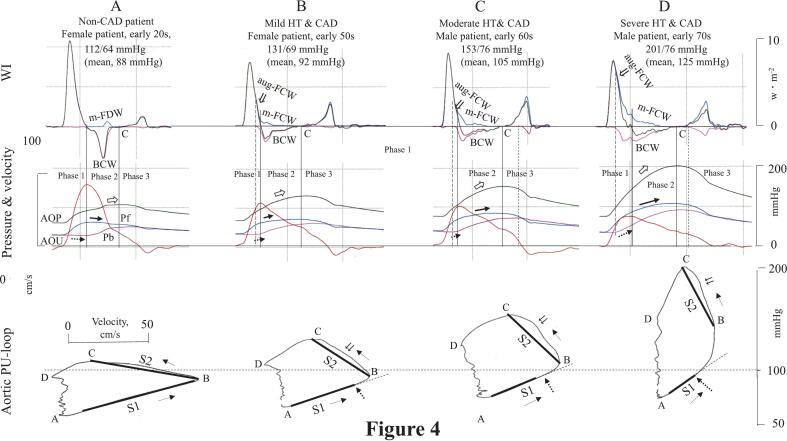
Comparisons of central aortic wave intensity waveforms (top panel), aortic pressure and flow velocity (middle panel), and the respective aortic pressure-velocity loop (bottom panel) in one control and three patients with CAD with differing HT severity The times of each waveform in the top and middle panels are matched. CAD, coronary artery disease; HT, hypertension; m-FCW, midsystolic forward compression wave. Other abbreviations are the same as in Fig. 1.

Patients with more severe HT showed steeper and shorter S1 slopes than the non-CAD patient because of the earlier onsets of BCW (Fig. 4). In the non-CAD patient (Fig. 4A), the forward and backward WI waves overlapped minimally because of the later onset of BCW in phase 2, which preserved the linearity and flatness of both the A-B (S1) and B-C (S2) lines and caused a slight decrease in P_f_ in phase 2 (bold arrow) with no apparent increase in AOP (open arrow).

In the hypertensive patients with CAD (Fig. 4B-D), the early onset of BCW in phase 1 induced an aug-FCW (⇓), bending the A-B line upward and increasing P_b_ (dashed arrow). The earlier the onset of the BCW, the greater the separation between the cumulative WI of the aug-FCW and the initial positive net-WI, and the greater the upward bending of the A-B line. The aug-FCW then continued into phase 2 as the m-FCW, opposing the BCW. The simultaneous presence of the m-FCW and BCW further increased AOP (open arrow) due to their combined compression effects. Because the onset of the m-FCW increased U, it offset the BCW-induced decrease in U in phase 2, making S2 steeper and convex toward the U axis (⇊ in Fig. 4B-D). The larger the relative magnitude of the m-FCW, the steeper S2 became. Although the relative magnitude of the m-FCW was too small to quantify by WIA, it can be identified through the change in S2.

### 3.4 Comparison of wave parameters between groups

Although no significant intergroup differences were found in the peak or cumulative WI of the BCW, the relative magnitude of the BCW was significantly higher in the CAD group (*P* = 0.015), as were the peak WI, cumulative WI, and relative magnitude of the m-FCW (*P* < 0.001) (Table 2). In contrast, the cumulative WI of the initial positive net-WI (∫ initial net-WI, *P* = 0.004); the peak WI, cumulative WI, and relative magnitude of the m-FDW; and %Td were higher in the non-CAD group (*P* < 0.001). The values of S1, S2, S1 + S2, and S2:S1 ratio were higher in the CAD group (S1: *P* = 0.013; others: *P* < 0.001). Among the wave parameters, the relative magnitude of the m-FCW showed the largest intergroup difference (0.01 in the non-CAD groups vs. 0.07 in the CAD group), with an effect size of *r* = 0.77.

**Table 2:** Comparison of wave parameters between groups.

Parameter	Overall (n = 31)	Non-CAD group (n = 10)	CAD group (n = 21)	*P* value	Effect size: *r*
**Wave intensity**					
**Peak** (10^4^·W·m^−2^·s^−2^)					
**Net WI**	**7.7 [4.8, 11.5]**	**10.8 [8.4, 17.3]**	**6.1 [4.5, 8.6]**	**0.013**^a^	0.44
BCW	1.7 [1.0, 2.7]	2.2 [1.0, 4.2]	1.6 [1.0, 2.4]	0.201	0.24
**m-FCW**	**0.4 [0.2, 0.7]**	**0.2 [0.1, 0.3]**	**0.6 [0.3, 1.2]**	**<0.001**^a^	0.56
**m-FDW**	**0.2 [0.1, 0.4]**	**0.5 [0.3, 1.4]**	**0.1 [0.1, 0.2]**	**<0.001**^a^	0.65
l-FDW	2.8 [2.0, 3.4]	2.9 [2.3, 4.0]	2.8 [1.9, 3.3]	0.519	0.12
**Cumulative** (10^2^·W·m^−2^·s^−1^)					
**∫ Initial net WI**	**24.7 [16.5, 35.2]**	**36.1 [28.1, 50.9]**	**20.9 [15.1, 27.9]**	**0.004**^a^	0.51
∫ BCW	10.1 [4.8, 11.8]	11.1 [4.1, 13.9]	9.0 [5.5, 13.0]	0.546	0.11
**∫ m-FCW**	**0.8 [0.2, 2.1]**	**0.2 [0.1, 0.2]**	**1.4 [0.7, 2.9]**	**<0.001**^a^	0.76
**∫ m-FDW**	**0.3 [0.1, 1.1]**	**1.6 [1.0, 3.4]**	**0.2 [0.1, 0.3]**	**<0.001**^a^	0.67
∫ l-FDW	9.1 [6.9, 11.8]	9.4 [6.8, 16.7]	9.1 [7.3, 11.2]	0.603	0.10
**Relative magnitude**					
**∫ BCW/ ∫ initial net WI**	**0.39 [0.28, 0.46]**	**0.29 [0.21, 0.37]**	**0.41 [0.33, 0.55]**	**0.015**^a^	0.43
**∫ m-FCW/ ∫ initial net WI**	**0.04 [0.01, 0.12]**	**0.01 [0.00, 0.01]**	**0.07 [0.03, 0.16]**	**<0.001**^a^	0.77
**∫ m-FDW/ ∫ initial net WI**	**0.01 [0.01, 0.04]**	**0.04 [0.03, 0.07]**	**0.01 [0.01, 0.01]**	**<0.001**^a^	0.68
**∫ l-FDW/ ∫ net WI**	**0.39 [0.27, 0.46]**	**0.30 [0.23, 0.38]**	**0.44 [0.36, 0.50]**	**0.005**^a^	0.49
**%Td**	**0.75 [0.63, 0.84]**	**0.90 [0.83, 0.96]**	**0.70 [0.54, 0.76]**	**<0.001**^a^	0.68
**PU-loop**					
**PWS (m/s)**	**7.6 [5.9, 11.9]**	**5.8 [5.0, 8.4]**	**9.5 [7.2, 12.3]**	**0.013**^a^	0.44
**S1 (m/s)**	**8.0 [6.2, 12.5]**	**6.0 [5.2, 8.9]**	**10.0 [7.5, 12.9]**	**0.013**^a^	0.44
**S2 (m/s)**	**13.5 [5.8, 21.4]**	**4.1 [2.4, 6.1]**	**16.4 [12.8, 26.3]**	**<0.001**^a^	0.72
**S1** **+** **S2**^b^**(m/s)**	**23.0 [13.4, 30.2]**	**10.1 [7.5, 15.3]**	**28.4 [20.6, 37.9]**	**<0.001**^a^	0.65
**S2:S1**^b^	**1.35 [0.76, 2.42]**	**0.53 [0.43, 0.82]**	**1.88 [1.29, 2.56]**	**<0.001**^a^	0.77

Data are shown as median [interquartile range: 25th percentile, 75th percentile].

%Td, time between the onset of the initial positive net-WI and the onset of the BCW divided by the ventricular acceleration time; BCW, backward compression wave; l-FDW, late forward decompression wave; m-FCW, midsystolic forward compression wave; m-FDW, midsystolic forward decompression wave; PU-loop, pressure-velocity loop; PWS, local aortic pulse wave speed; S1, initial linear part of the A-B ascending slope of the aortic PU-loop; S2, slope of the loop's second B-C ascending limb from the peak velocity point (B) to the peak aortic pressure point (C); WI, wave intensity; ∫ initial net WI, cumulative initial positive net wave intensity.

a Significant *P* values (*P* < 0.05) are written in bold.

b S1 + S2 and the S1:S2 ratio were calculated using the absolute values of S1 and S2.

### 3.5 Comparison of changes in P_b_ and P_f_ during systole between groups

In phase 1, P_b_ decreased in the non-CAD group but increased in the CAD group (*P* = 0.002). In phase 2, P_b_ increased less in the non-CAD group than in the CAD group (*P* = 0.019), whereas P_f_ decreased in the non-CAD group but increased in the CAD group (*P* < 0.001) (Table 3). Notably, P_f_ exhibited opposite directional changes between the two groups in phase 2 (−6.2 in the non-CAD group vs. +8.8 in the CAD group), representing the most pronounced intergroup difference, with the largest effect size (*r* = 0.74).

**Table 3:** Changes in backward- and forward-traveling aortic pressure components during systole.

Component	Overall (n = 31)	Non-CAD group (n = 10)	CAD group (n = 21)	*P* value	Effect size: *r*
Phase 1					
**P**_**b**_**, mmHg**	**2.2 [−0.4, 3.9]**	**−1.9 [−4.5, 1.8]**	**2.4 [1.6, 4.5]**	**0.002**^a^	0.54
P_f_, mmHg	32.9 [29.4, 39.5]	38.2 [31.5, 42.0]	31.8 [27.3, 37.3]	0.105	0.29
Phase 2					
**P**_**b**_**, mmHg**	**23.4 [19.2, 28.8]**	**19.9 [16.4, 23.9]**	**25.2 [20.5, 30.9]**	**0.019**^a^	0.42
**P**_**f**_**, mmHg**	**3.1 [−4.6, 13.1]**	**−6.2 [−11.4, −3.6]**	**8.8 [2.1, 13.7]**	**<0.001**^a^	0.74

Data are shown as median [interquartile range: 25th percentile, 75th percentile].

Phase 1, ventricular acceleration phase; phase 2, midsystole between the end of the first and the onset of the second positive net wave intensity wave; P_b_, backward-traveling pressure; P_f_, forward-traveling pressure.

a Significant *P* values (*P* < 0.05) are written in bold.

### 3.6 Correlation analysis

The ∫ initial net-WI, which represents left ventricular acceleration, negatively correlated with local aortic PWS (*r* = −0.650, *P* < 0.001) (Table 4). It also correlated with %Td (*r* = 0.487, *P* = 0.005), which in turn negatively correlated with the relative magnitude of m-FCW (*r* = −0.480, *P* = 0.006) and local aortic PWS (*r* = −0.406, *P* = 0.023), but not with the relative magnitude of BCW (*r* = −0.306, *P* = 0.095). These results suggest that a decrease in ∫ initial net-WI may lead to earlier onset of the BCW (reflected by lower %Td) in the presence of increased local aortic PWS, which in turn triggers the onset of m-FCW rather than altering the relative magnitude of the BCW itself. The relative magnitude of the m-FCW correlated more strongly than that of the BCW with the phase 1 increase in P_b_ (*r* = 0.608, *P* < 0.001 vs *r* = 0.374, *P* = 0.038) and the phase 2 increases in P_b_ (*r* = 0.746, *P* < 0.001 vs *r* = 0.745, *P* < 0.001) and P_f_ (*r* = 0.838, *P* < 0.001 vs *r* = 0.646, *P* < 0.001), both of which should increase AOP and left ventricular workload. The relative magnitude of m-FDW correlated with both ∫ initial net-WI (*r* = 0.645, *P* < 0.001) and %Td (*r* = 0.624, *P* < 0.001), respectively.

**Table 4:** Correlations among WIA parameters, local aortic PWS, and phasic changes in P_b_ and P_f_.

	**∫** initial net WI		P_b_ in phase 1		P_f_ in phase 1		P_b_ in phase 2		P_f_ in phase 2		%Td	
	*r*	*P*	*r*	*P*	*r*	*P*	*r*	*P*	*r*	*P*	*r*	*P*
∫ initial net WI	–	–	**−0.364**	**0.044**	**0.425**	**0.017**	−0.024	0.898	**−0.435**	**0.014**	**0.487**	**0.005**
∫ BCW / ∫ net WI	−0.265	0.150	**0.374**	**0.038**	−0.193	0.299	**0.745**	**<0.001**	**0.646**	**<0.001**	−0.306	0.095
∫ m-FCW / ∫ net WI	−0.310	0.089	**0.608**	**<0.001**	−0.179	0.335	**0.746**	**<0.001**	**0.838**	**<0.001**	**−0.480**	**0.006**
∫ m-FDW / ∫ net WI	**0.645**	**<0.001**	**−0.528**	**0.002**	0.261	0.157	−0.138	0.459	**−0.602**	**<0.001**	**0.624**	**<0.001**
Local aortic PWS	**−0.650**	**<0.001**	**0.450**	**0.011**	−0.015	0.936	0.194	0.297	**0.383**	**0.033**	**−0.406**	**0.023**

*r*: correlation coefficient.

Significant *P* values (*P* < 0.05) are written in bold.

%Td, relative onset time of the backward compression wave; BCW, backward compression wave; m-FCW, midsystolic forward compression wave; m-FDW, midsystolic forward decompression wave; P_b_, backward-traveling component of aortic pressure; P_f_, forward-traveling component of aortic pressure; PU-loop, pressure-velocity loop; PWS, pulse wave speed; WIA, wave intensity analysis; ∫ initial net WI, cumulative initial positive net wave intensity.

### 3.7 Relationships between aortic PU-loop markers and relative magnitude of BCW and m-FCW

**Fig. 5:**
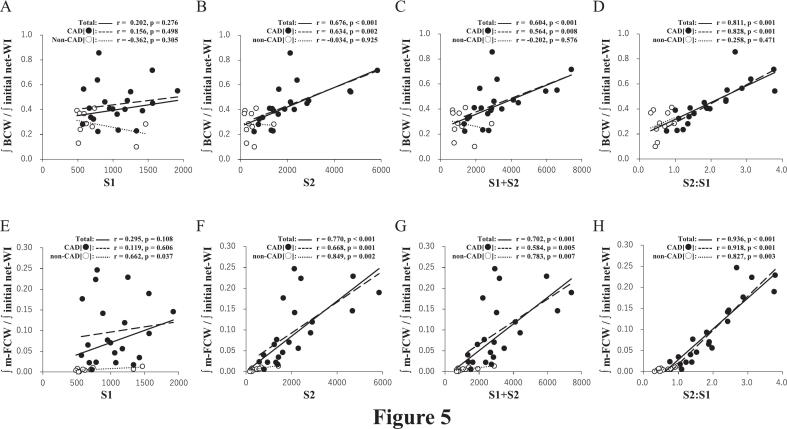
Correlations between loop markers and relative magnitude of BCW and m-FCW Relative magnitude of BCW, cumulative wave intensity of BCW divided by that of initial positive net wave intensity; relative magnitude of m-FCW, cumulative wave intensity of m-FCW divided by that of initial positive net wave intensity. Other abbreviations are the same as in Fig. 1.

The S2:S1 ratio of the aortic PU-loop had the strongest correlation with the relative magnitude of m-FCW (*r* = 0.936, *P* < 0.001) and had a stronger correlation than S2 and S1 + S2 with the relative magnitude of BCW (*r* = 0.811, *P* < 0.001) (Fig. 5). S1 had no correlation with the relative magnitude of either BCW or m-FCW.

### 3.8 ROC analysis of WIA and aortic PU-loop variables for identifying patients with CAD

**Fig. 6:**
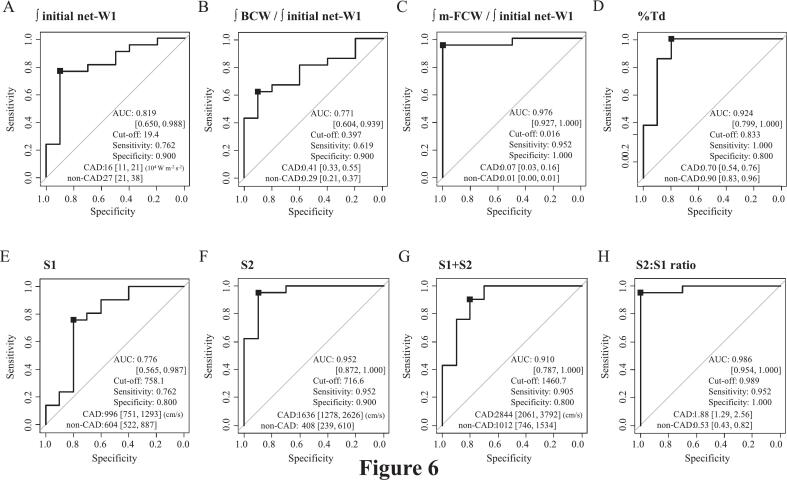
Receiver operating characteristic curve analyses of relevant variables for identifying CAD Receiver operating characteristic curves were generated to compare the area under the curve values of ∫ relevant variables for identifying patients with CAD. Black boxes define the cutoff values of each variable. AUC, area under the curve; BCW, backward compression wave; CAD, coronary artery disease; m-FCW, midsystolic forward compression wave; ∫ initial net-WI, cumulative wave intensity of the initial positive net wave intensity; %Td, time between the onset of the initial positive net wave intensity and the onset of the backward compression wave divided by the ventricular acceleration time. Other abbreviations are the same as in Fig. 1.

ROC curve analysis showed that the relative magnitude of m-FCW had higher accuracy (AUC = 0.976) than that of BCW (AUC = 0.771) for identifying patients with CAD (Fig. 6). The early onset of BCW, visible as the upward bending point of S1, was also more useful than the relative magnitude of BCW for identifying cardiovascular risk, with a decrease in %Td showing an AUC of 0.924. However, the S2:S1 ratio was the most effective marker for identifying patients with CAD, with a higher AUC (0.986) than S1 (0.776), S2 (0.952), S1 + S2 (0.910), and other variables obtained by WIA.

## 4 Discussion

### 4.1 Ventriculo-arterial interaction in the central aorta in non-CAD patients

Nobel demonstrated that left ventricular outflow continues into late systole under its own momentum, and that the aorta acts as a pressure source for the left ventricle [19]. Penny et al. observed an m-FDW in the aorta of sheep and suggested that it resulted from the aortic inertial effect and had a highly linear relationship with ∫ initial net-WI [20]. In the present study, ∫ initial net-WI was also significantly correlated with the relative magnitude of m-FDW. These findings suggest that adequate ∫ initial net-WI provides sufficient inertia for blood to keep flowing during midsystole with the onset of the m-FDW, without an apparent increase in P_f_ [21].

**Fig. 7:**
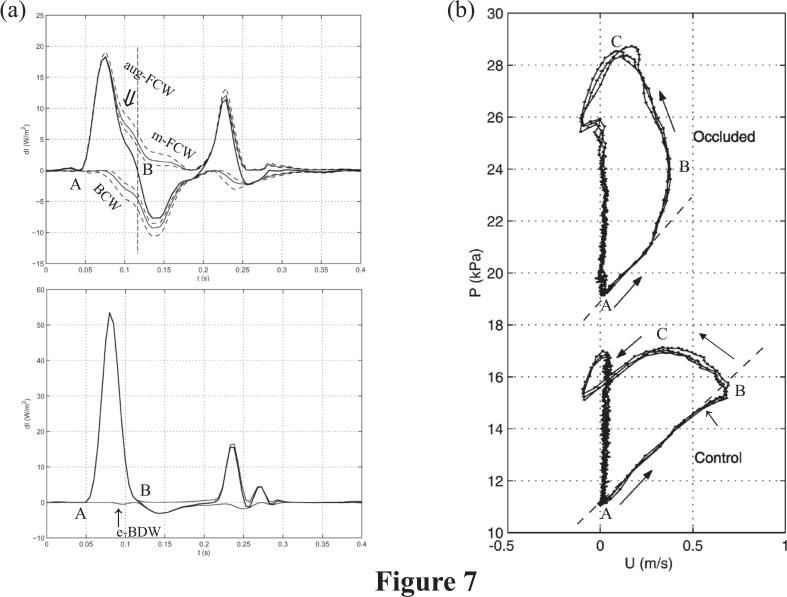
Changes in wave intensity waveforms and aortic pressure-velocity loops before and after total occlusion of the upper thoracic aorta in dogs a) The wave intensity waveform after (top) and before (bottom) total occlusion of the upper thoracic aorta in a dog. Note the development of the augmented forward compression wave (aug-FCW) followed by midsystolic forward compression wave (m-FCW, positive dashed lines) opposing the early onset of the backward compression wave (BCW) (negative dashed lines) before the end of the ∫ initial net wave intensity (solid line) after aortic occlusion. b) The aortic pressure-velocity loop after (top) and before (bottom) total occlusion of the aorta. Note the change from downward bending of the loop's initial ascending slope caused by a small early backward decompression wave (e-BDW; ↑) before occlusion to upward bending opposing the early onset of the BCW after occlusion. Reproduced with permission from Khir AW, O'Brien A, Gibbs JSR, Parker KH. Journal of Biomechanics 2001;34(9):1145–1155. © Elsevier. RightsLink order/license #6103901069974. DOI:https://doi.org/10.1016/S0021-9290(01)00076-8

Penny et al. also reported an aortic e-BDW in sheep and suggested that it reflected a functional increase in aortic cross-sectional area during systole [20]. The central aorta is the main determinant of total systemic arterial compliance and is responsible for the Windkessel buffering effect [22]. The onset of a small e-BDW that induced downward bending of the PU-loop was also observed by Khir et al. at steady state in the central aorta in dogs (↑; Fig. 7, bottom) [18]. In the present study, the e-BDW observed in the youngest non-CAD patient may reflect this buffering effect of the central aorta, which may enhance blood acceleration and subsequent inertia [23].

### 4.2 Ventriculo-arterial interaction in the central aorta in patients with CAD

Li et al. [12] reported that aging is associated with decreased ∫ initial net-WI and increased local central aortic PWS in healthy individuals, resulting in earlier BCW onset in an MRI study. In our study, the CAD group also showed earlier BCW onset, as indicated by the decrease in %Td, than the non-CAD group. Additionally, %Td positively correlated with ∫ initial net-WI and negatively correlated with local central aortic PWS and relative magnitude of m-FCW, both of which were significantly higher in the CAD group. These results suggest that in the CAD group, the insufficient both ∫ initial net-WI and aortic buffering effect due to increased local central aortic PWS provoked the onset of the m-FCW as a compensatory mechanism to support blood inertia in response to the early BCW. Thus, the onset of the m-FCW may reflect a state in which midsystolic flow is maintained by persistent myocardial shortening that matches the relative magnitude of the BCW with an increase in Pf. Consistent with this interpretation, the intergroup difference in Pf during phase 2 was the most pronounced among the hemodynamic parameters, with opposite directional changes observed between the non-CAD and CAD groups. If the ∫ initial net-WI and aortic buffering effect are sufficient to maintain blood inertia during midsystole, the BCW develops later, and the m-FCW does not occur with a decrease in Pf. Therefore, m-FCW may be considered a key pulse wave that represents impaired ventriculo-arterial interaction. In the present study, the median and 25th and 75th percentile values of the relative magnitude of m-FCW showed the greatest difference between the non-CAD group (0.01 [0.00, 0.01]) and the CAD group (0.07 [0.03, 0.16]) among variables related to ventriculo-arterial interaction (effect size: *r* = 0.77, *p* < 0.001).

Khir et al. used WIA in dogs to demonstrate that occlusion of the central aorta, which decreased ∫ initial net-WI and increased local aortic PWS, provoked an aug-FCW followed by an m-FCW in response to the early BCW with an increase in AOP. (Fig. 7, top) [18]. Thus, the relative magnitude of m-FCW could be used to evaluate the adequacy of ventriculo-arterial interaction.

The generation of the m-FCW, even at relatively low values, may have a deleterious effect on cardiac performance by impaired ventriculo-arterial interaction, especially in atherosclerotic CAD patients. This may explain why the relative magnitude of m-FCW showed better diagnostic performance than that of BCW for identifying patients with CAD (AUC = 0.976 vs 0.771) in ROC analysis.

### 4.3 Ventriculo-arterial interaction in the central aorta in patients with HT

Generation of the m-FCW via altered ventriculo-arterial interaction increases AOP, and sustained AOP elevation may increase arterial stiffness, which plays a role in the development of HT. HT, in turn, is a risk factor for atherosclerosis and may increase left ventricular workload and impair cardiac performance by decreasing ∫ initial net-WI. This decrease may further contribute to m-FCW generation, thereby creating a vicious cycle. Thus, m-FCW generation may be the initial trigger of this cycle, leading to HT and CAD. In the present study, HT was present in 17 out of 21 CAD patients (81%). Substantial evidences indicate that HT promotes atherosclerosis and increases the likelihood of CAD [24], [25], [26]. Thus, the magnitude of the m-FCW may also serve as a marker for identifying patients with hypertensive cardiovascular risk in primary prevention.

Further studies on the biomechanics and cellular mechanisms of the m-FCW could provide a better understanding of the pathophysiology of cardiovascular risk in patients with atherosclerotic CAD.

### 4.4 Effects of ∫ initial net-WI, local aortic PWS, and wave reflections on the aortic PU-loop

This study demonstrated that aortic properties and ventriculo-arterial interaction are reflected in characteristic features of the PU-loop. Specifically, S1 and S2 both represent the increase in P required to sustain U at a given point in systole and thus reflect ventriculo-arterial interaction. In the non-CAD group, midsystolic flow is primarily maintained by inertia, accompanied by a decrease in P_f_. Accordingly, S1 remained low, and S2 did not exceed S1 (S1 > S2), with no apparent development of the m-FCW. In contrast, in the CAD group, an increase in P_f_ indicates insufficient blood inertia to maintain midsystolic flow. Under these conditions, S2 increased and exceeded S1 (S1 < S2), reflecting the extent to which the m-FCW compensates for this deficit. In other words, within the PU-loop framework, S1 is proportional to local pulse wave speed and therefore reflects the inertial load imposed by aortic stiffness. By contrast, S2 reflects the degree of the compensatory m-FCW that emerges when blood inertia is insufficient to sustain midsystolic flow. Consequently, the S2:S1 ratio could serve as a marker of impaired ventriculo-arterial interaction and could potentially identify atherosclerotic CAD with high cardiovascular risk. Consistent with this interpretation, it had a higher AUC (0.986) than the other loop markers for identifying CAD. Furthermore, the median and 25th and 75th percentile values of the S2:S1 ratio in the CAD group (1.88 [1.29, 2.56]) were significantly higher than in the non-CAD group (0.53 [0.43, 0.82]), and this ratio showed the strongest correlation with the relative magnitude of m-FCW (*r* = 0.936, *P* < 0.001). The optimal cutoff value for identifying patients with CAD was 0.989, as determined by the maximum Youden index.

In dogs, Khir et al. also demonstrated that the downward bending of the aortic A-B line, induced by a small e-BDW (↑), was replaced by upward bending, along with steepening of the B-C slope (S2), after aortic clamping (Fig. 7) [18]. Clamping the aorta resulted in a much greater increase in the B-C slope than in the A-B slope (S2 **>** S1), along with an obvious decrease in ∫ initial net-WI, reflecting impaired inertia (Fig. 7). These loop shapes before and after clamping closely resemble those observed in our youngest non-CAD patient and patients with atherosclerotic CAD, respectively.

Noninvasive measurement of central aortic pressure remains challenging. Aortic diameter (D), derived from cross-sectional area measurements using phase contrast MRI, have been used as a surrogate for central aortic pressure [12]. An advantage of this method is that phase images obtained with the same MRI sequence provide simultaneous flow information, and WIA using the DU-loop has been proposed for assessing ventriculo-arterial interaction. However, even slight differences between D and P may be amplified by the complex calculations required for WIA, potentially reducing reliability. Therefore, it may be difficult to accurately quantify the m-FCW, which accounts for only 0.07% of the initial net-WI, using a noninvasive WIA. In contrast, an index derived from a loop obtained by simply plotting D against U, without complex processing, is more likely to retain sufficient accuracy. In fact, local PWS in the central aorta has been assessed using the DU-loop, and the early onset of BCW has been also clearly identified by the upward bending point of the DU-loop (Fig. 1) [12].

Coronary microvascular disease (CMD) is a major contributor to CIHD [27]. Diffuse non-obstructive atherosclerosis of the epicardial coronary arteries is found in the majority of symptomatic patients with CMD [28], [29]. The epicardial obstructive coronary artery stenosis observed on coronary angiography in the CAD group in this study likely represents the end-stage manifestation of systemic atherosclerosis. Therefore, the S2:S1 ratio derived from the aortic PU-loop may also have strong potential for differentiating high-risk CMD patients with chronic non-obstructive atherosclerotic disease having no obvious angiographic stenosis from those with other non-atherosclerotic CIHD [30], [31].

### 4.5 Limitations and perspectives

This study has several limitations. First, the clinical diagnosis of CAD was made by routine clinical tests, including stress tests and echocardiography. However, because reliable non-invasive diagnostic methods for myocardial ischemia (such as coronary computed tomography and cardiac MRI) were not yet established during the study period, we cannot rule out the possibility that some patients with non-cardiac chest pain were included in the non-CAD group. Second, as it was retrospective and based on a limited dataset, we were unable to use age-matched non-CAD and CAD patients, limiting comparisons between the two groups. Third, the inclusion of patients with HT in the non-CAD group may have biased the results, because these patients are likely to have more advanced atherosclerosis than other non-CAD patients. Regardless, age and HT play major roles in increasing aortic stiffness and wave reflections, and it is reasonable that the CAD group included a significantly higher number of elderly and hypertensive patients. Thus, these limitations do not affect the overall findings of the present study, which aimed to examine the usefulness of the aortic PU-loop as a potential diagnostic tool to differentiate high-risk CAD from other CIHD. Fourth, to verify the reproducibility of the WIA values obtained, prospective validation studies are needed to link wave parameters to atherosclerotic burden or clinical outcomes. However, as simultaneous high-fidelity P and U measurement at the same site in the central aorta is essential for detailed WIA, we currently lack appropriate alternative tools to noninvasively measure the extremely small m-FCW signal, which represents only approximately 0.07% of the ∫ initial positive net-WI. Therefore, further prospective studies are warranted to evaluate the potential of the aortic PU-loop using an advanced noninvasive method in a larger number of CIHD patients.

## 5 Conclusions

The insufficient blood inertia during ventricular acceleration may trigger a previously unreported m-FCW via impaired ventriculo-arterial interaction, potentially increasing cardiovascular risk. The magnitude of blood inertia and the m-FCW are captured by the initial linear slope (S1) and the second ascending slope (S2), respectively, of the aortic PU-loop. The S2:S1 ratio may serve as a potential diagnostic marker of impaired ventriculo-arterial interaction, thereby helping assess a patient's cardiovascular risk. This marker may be particularly helpful in distinguishing CAD with systemic atherosclerosis from other CIHD with different causes of myocardial ischemia.

## CRediT authorship contribution statement

**Shizuo Hanya:** Writing – review & editing, Writing – original draft, Visualization, Methodology, Investigation, Formal analysis, Data curation, Conceptualization. **Hajime Yamakage:** Writing – review & editing, Formal analysis, Data curation. **Yukiyoshi Kondoh:** Investigation. **Motoaki Sugawara:** Writing – review & editing, Formal analysis.

## Ethical statement

This study was conducted in accordance with the Declaration of Helsinki and was approved by the Ethics Committee of Tokyo Women's Medical University. Written informed consent was obtained from all participants prior to inclusion in the study. All procedures were carried out in compliance with relevant laws and institutional guidelines.

## Funding

This work was supported by the 10.13039/501100013642Japan Heart Foundation and JSPS KAKENHI grants.

## Declaration of competing interest

The authors declare that they have no known competing financial interests or personal relationships that could have appeared to influence the work reported in this paper.
